# (-)-Epigallocatechin-3-gallate Ameliorates Intervertebral Disc Degeneration Through Reprogramming of the Circadian Clock

**DOI:** 10.3389/fphar.2021.753548

**Published:** 2021-11-04

**Authors:** Liangwei Mei, Yi Zheng, Teng Ma, Bing Xia, Xue Gao, Yiming Hao, Zhuojing Luo, Jinghui Huang

**Affiliations:** ^1^ Department of Orthopaedics, Xijing Hospital, the Fourth Military Medical University, Shaanxi, China; ^2^ Faculty of Life Sciences, Northwest University, Shaanxi, China

**Keywords:** circadian clock, intervertebral disc degeneration (IDD), (-)-epigallocatechin-3-gallate (EGCG), oxidative stress, Bmal1

## Abstract

The circadian clock is vital in the management of our daily physiological as well as metabolic processes. Disturbances of the clock can cause degenerative and age-related diseases. Increasing evidence has indicated that the intervertebral discs contain an internal biological clock related to degeneration. However, to date, no bioactive compounds have been found that can ameliorate intervertebral disc degeneration (IDD) by restoring the circadian clock. (-)-Epigallocatechin-3-gallate (EGCG) is a nutritious food with powerful antioxidant properties, as well as entraining biological clock to improve health. The purpose of this study was to determine whether the protective effects of EGCG on nucleus pulposus (NPCs) under oxidative stress is related to the circadian clock. First, we found that EGCG attenuated H_2_O_2_-induced extracellular matrix degradation in NPCs and inhibited H_2_O_2_-induced NPC apoptosis. Our *in vivo* experiments also confirmed this finding. Furthermore, EGCG attenuated H_2_O_2_-triggered dampening of phase shifts and daily oscillations in circadian clock gene transcription as well as protein expression levels. Intriguingly, core clock gene (Bmal1) knockdown notably blocked the protective effects of EGCG. To our knowledge, this study provides the first convincing evidence that EGCG prevents IDD in a Bmal1-dependent manner. In general, EGCG supplementation can be used as a nutritional prevention strategy for the rehabilitation of degenerative diseases related to the circadian clock.

## Introduction

Due to Earth’s rotation, almost all living species have evolved an internal timing system referred to as the circadian clock that enables adaptation to changes in behavioral as well as physiological aspects of the daily environment (Ruan et al., 2021). The circadian clock of mammals consists of the main pacemaker in the suprachiasmatic nucleus (SCNs) of the hypothalamus, which is synchronized with auxiliary peripheral oscillations that are found in almost all body cells (Harvey et al., 2020). At the molecular level, dependent on core clock circuit interlock transcription - translation of a feedback loop, these loops drive the core clock as well as clock control routine oscillation genes, such as circadian locomotor output cycles kaput (Clock), brain and muscle aryl hydrocarbon receptor nuclear translocator-like1 (Bmal1), period genes (Per1 and Per2), as well as cryptochrome genes (Cry1 and Cry2) (Patke et al., 2020). Disturbances of circadian oscillator components and abnormal or defect synchronization of the circadian clock pathway can cause circadian rhythm disorders, which are associated with high risks for diabetes, degenerative diseases, and cancer (Kinouchi and Sassone-Corsi, 2020; Peek, 2020; Morris et al., 2021).

Accumulating evidence suggests that there is a close correlation between the circadian clock and oxidative stress. Cellular redox state is critically important for the regulation of the Bmal1 and Clock genes transcriptional activities (Ranieri et al., 2015). On the other hand, circadian clock involves in the regulation of reactive oxygen species (ROS) levels *in vitro* and *in vivo* (Liu et al., 2021). Night-shift work as well as frequent travel across time zones disrupts the circadian clock and can significantly increase the levels of oxidative stress markers (Ozyurek et al., 2021). As a core gene of the circadian clock, Bmal1 plays a role in regulation of tissue homeostasis by directly controlling ROS levels. In Bmal1^−/−^ mice, it was found that they exhibited significantly increased kidneys, heart, as well as spleen ROS levels (Kondratov et al., 2009). Specific knockout of Bmal1 in mouse islet *β* cells significantly improves the ROS sensitivity of fibroblasts (Lee et al., 2018). In summary, the circadian clock mediates variations in cellular redox tone by regulating redox pathway expression. Since many aspects of oxidative stress and aging are closely related, age-associated alterations in clock function can promote oxidative-related damage.

Recently, a self-regulating circadian clock was discovered in intervertebral discs (IVDs), and its disappearance led to IVD degeneration (IDD) (Dudek et al., 2017; Sherratt et al., 2019; Zhang et al., 2020; Morris et al., 2021). Molecular clock genetic disruption in IVD mice models, particularly through Bmal1 disruption, enhanced mice susceptibility to IDD (Dudek et al., 2017). Another study confirmed that passive smoking altered circadian clock genes in IVD rat models, with a majority of the genes exhibiting a phase shift of between −6 to −9 h and some clock-associated genes exhibiting oscillation disappearance in the nucleus pulposus (NP) tissue (Numaguchi et al., 2016). In addition, Bmal1 and retinoic acid receptor-related orphan receptor (ROR)-α regulate the activity of hypoxia-inducible factor (HIF)-1 in NP cells (NPCs), and is involved in NPC adaptation to their hypoxic niches. Imbalances in these proteins affect normal tissue homeostasis as well as function (Suyama et al., 2016). These findings emphasize the essential role of the circadian clock in maintaining IVD homeostasis. Therefore, evaluation of therapeutic efficacies of clock-targeting compounds for correcting circadian clock dysfunction and misalignment, and hence attenuate the degradation process will be a new research field (Ribeiro et al., 2021).

(-)-Epigallocatechin-3-gallate (EGCG), a catechin that is the most biologically active component in green tea, has antioxidant, anticancer, anti-inflammatory, as well as cardioprotective biological activities (Chakrawarti et al., 2016). Although the possible toxicity and rare cases of liver injury has been reported after consumption of green tea infusions, it is critical to find the appropriate dose at which EGCG could bring more health benefits with lower toxicity (EFSA Panel on Food Additives and Nutrient Sources added to Food (ANS) et al., 2018; Ouyang et al., 2020). In the hypothalami of diet-induced obese (DIO) mice models, EGCG treatment increased the expression levels of key circadian clock genes, Clock and Bmal1 (Li et al., 2016). EGCG eliminated high-fat/fructose diet (HFFD)-induced circadian asynchrony and metabolic-associated disorders in C57BL/6 mice (Mi et al., 2017a). EGCG also exhibits Bmal1-dependent effects on insulin resistance (Mi et al., 2017b). This evidence suggests that EGCG could improve health through entraining circadian rhythms.

Previous studies have shown that EGCG inhibits IDD through multiple signaling pathways (Krupkova et al., 2014; Tian et al., 2020). In our study, we propose the hypothesis that whether circadian clock-associated mechanisms play a role in preventive effects of EGCG against IDD. We evaluated the importance of the circadian regulator (Bmal1) in potential effects of EGCG on the pathological process of IDD. Moreover, we examined the protective activities of EGCG on hydrogen peroxide (H_2_O_2_)-induced oxidative stress in NPCs. Our findings inform future clinical applications of EGCG as a novel therapeutic strategy for degenerative disease through circadian clock-related mechanisms.

## Materials and Methods

### Chemicals and Reagents

EGCG and H_2_O_2_ were bought from Sigma-Aldrich (St. Louis, MO, United States). The Dulbecco’s modified Eagle’s medium/nutrient mixture F-12 (DMEM/F12) was purchased from Jet Biofil (Guangzhou, China), and fetal bovine serum (FBS) was procured from Gibco-BRL (Gaithersburg, MD, United States). OptiVitro^®^ serum-free cell cryopreservation medium was purchased from Excell (Excell Bio, China). Cell culture plate was purchased from *In Vitro* Scientific (Hangzhou Xinyou Biotechnology Co., Ltd., China). A Cell Counting Kit-8 (CCK-8) assay kit was purchased from MultiSciences (Hangzhou, China). PBS was purchased from HAKATA (Shanghai Chuanqiu Biotechnology Co., Ltd, China). The primary antibodies against Bmal1, Aggrecan, Collagen II, matrix metalloproteinase 13 (MMP13), ADAM metallopeptidases with thrombospondin type 1 motif (ADAMTS5), Bcl-2, Bax, and GAPDH were obtained from Abcam (Cambridge, United Kingdom). Primary antibodies against cleaved caspase3 were procured from Cell Signaling Technology (Danvers, MA, United States).

### Isolation and Culture of Rat NPCs

NP tissues were obtained from lumbar IVD of male Sprague-Dawley rats (4-week-old). Then, tissues were sliced and treated for 4 h with collagenase II (0.025%; Gaithersburg, MD, United States) at 37°C. NPC cultures were done in DMEM/F12 medium with 10% FBS and incubated at 37°C in a humidified 5% CO_2_ environment. Cultured medium was changed every 2 days. Cells from 3–5 passages were used in experiments.

### Cell Viability Assays

Cytotoxic effects of EGCG or H_2_O_2_ on NPCs were determined by CCK-8 assay. NPCs were seeded in 96-well plates (6 × 10^3^ cells/well) containing complete DMEM/F12 medium. After 24 h, cells were subjected to EGCG treatment at different doses (0, 10, 20, 40, 60, 80, or 100 μM) or H_2_O_2_ (0, 100, 200, 300, or 400 μM) for 24 h. Then, the CCK-8 reagent (10 μl) was added to each well followed by incubation for 1 h in dark. Absorbance was determined at 450 nm using a microplate reader (ELX800; Bio-Tek, Winooski, VT, United States).

### Quantitative Real-Time Polymerase Chain Reaction (QPCR) Assay

Total RNA were extracted by a TaKaRa MiniBEST Universal RNA Extraction Kit (Takara Bio, Kyoto, Japan) as instructed by the manufacturer. Then, reverse transcription and amplification of the extracted RNA was done by One-Step qRT-PCR TB Green^®^ Kit (Takara, Kyoto, Japan). Establishment of a stable, reliable standard curve was done by plotting threshold cycle (Ct) values. We chose GAPDH (Sino Biological Inc., Beijing, China) as the internal control. Relative mRNA levels were calculated by the 2^−ΔΔCt^ method. Table 1 shows the primers used in this study.

**TABLE 1:** The rat primer sequences used in qPCR.

Gene	Stream	Sequence (5–3′)
Bmal1	Forward	TGG​CCA​GAG​TGA​ATG​CTT​TTG
	Reverse	CCT​GAC​TGG​CCT​GGA​ACT​TG
Clock	Forward	TGC​TGT​CCT​TAC​TGC​TTG​GT
	Reverse	TGG​CAA​AGT​GGT​GAT​ACC​TGA
Per1	Forward	GTG​CTC​CAG​GAT​CCC​ATC​TG
	Reverse	CTC​TGA​GAA​CCG​TGG​CTG​TT
Per2	Forward	GAC​CAT​CTG​TGT​GTC​ATC​TGG​A
	Reverse	CTC​ATG​TGG​CTT​CCC​CGT​TA
Cry1	Forward	TGC​GCA​TTT​CAC​ACA​CAC​TG
	Reverse	GAC​AGA​GGG​GTT​GTG​CAC​TT
Cry2	Forward	TAG​AAC​CCG​CAG​CAG​TAA​CC
	Reverse	GAC​ACA​TCT​ATT​CCA​GCC​TGC
Collagen II	Forward	CAC​GCC​TTC​CCA​TTG​TTG​AC
	Reverse	CCG​GAC​TGT​GAG​GTT​AGG​ATA​G
Aggrecan	Forward	CAG​ATG​GCA​CCC​TCC​GAT​AC
	Reverse	ACA​CAC​CTC​GGA​AGC​AGA​AG
MMP13	Forward	ACC​ATC​CTG​TGA​CTC​TTG​CG
	Reverse	TTC​ACC​CAC​ATC​AGG​CAC​TC
ADAMTS5	Forward	GTC​CAA​ATG​CAC​TTC​AGC​CAC​GAT
	Reverse	AAT​GTC​AAG​TTG​CAC​TGC​TGG​GTG
GAPDH	Forward	TTG​TAA​CCA​ACT​GGG​ACG​ATA​TGG
	Reverse	GAT​CTT​GAT​CTT​CAT​GGT​GCT​AGG

### Western Blot Assay

NPCs were cultured in 6-well plates containing complete medium and incubated to cell densities of ∼90%. EGCG was used to pretreat the cells for 4 h, followed by stimulation with H_2_O_2_ for 24 h. Cells were lysed using the RIPA lysis buffer to obtain total proteins. Extracted proteins were separated by sodium dodecyl sulfate-polyacrylamide gel electrophoresis (SDS-PAGE) with 12% gel and later transferred to polyvinyldifluoride (PVDF) membranes. Skim milk (5%, dissolved in TBST) was used to block the membranes for 1 h at 37°C. After overnight incubation with primary antibodies at 4°C, membranes were incubated for 1 h with goat anti-rabbit HRP-conjugated antibody (*70-GAR007*, MultiSciences, Hangzhou, China) and visualized using an Amersham Imager 600 (GE Healthcare, Amersham, United Kingdom).

### Immunofluorescence Staining

NPCs were fixed for 20 min in paraformaldehyde (4%) and permeabilized with Triton X-100 (0.1%) for 15 min. Then, cells were blocked for 1 h using QuickBlock Blocking Buffer for Immunol Staining (Beyotime, Shanghai, China) followed by overnight incubation in the presence of primary antibodies against Collagen II (1:200) and MMP13 (1:500) at 4°C. Then, cell incubation was done for 2 h with appropriate FITC-conjugated secondary antibodies (1:400) at 37°C and finally incubated for 5 min with 4′,6-diamidino-2-phenylindole (DAPI) (Sangon Biotech), followed by fluorescence microscopy.

### Terminal Deoxynucleotidyl Transferase-Mediated dUTP Nick-End Labeling (TUNEL) Staining

To examine apoptosis in each group, staining of NP cells or tissues was done using a One Step TUNEL Apoptosis Assay Kit (Beyotime, Shanghai, China), as described the manufacturer.

### Small Interfering RNA Transfection of NPCs

Transfection of NPCs in 6-well plates was done with control siRNA (si-ctrl; sense 5′-UUC​UCC​GAA​CGU​GUC​ACG​UTT-3′, antisense 5′-ACG​UGA​CAC​GUU​CGG​AGA​ATT-3′) or Bmal1 siRNA (si-Bmal1; sense 5′-GGC​ACA​UCG​UGU​UAU​GAA​UTT-3′, antisense 5′-AUU​CAU​AAC​ACG​AUG​UGC​CTT-3′) using a Lipofectamine 2000 transfection reagent (Thermo Fisher Scientific, Waltham, MA, United States). Then, after transfection with siRNA for 48 h, assessments of transfection efficiencies were done by Western blot and qPCR analyses.

### IDD Animal Model

The animal experimental protocols were permitted by the Ethics Committee of Xijing Hospital, the Fourth Military Medical University. Thirty-six male Sprague-Dawley rats (4 months in age) were used in this study. Animals were kept under standard light-dark cycle (12-h light/dark cycle) and temperature (22 ± 2°C) conditions. Rats were randomized into three groups of 12 each (control, IDD and IDD + EGCG groups). To induce anesthesia, 10% chloral hydrate (3.6 ml/kg) was intraperitoneally (i.p.) administered. The IDD and IDD + EGCG groups were subjected to IVD puncture surgery using a 27-gauge needle in the Co7/8 disc (Lyu et al., 2021). After puncture, EGCG dissolved in NaCl solution (0.9%) was intragastrically administered to rats in the IDD + EGCG group three times a week using a syringe (50 mg/kg body weight). The IDD group rats were given an equal amount of saline solution. The dose of EGCG was determined based on previous reports (Cai et al., 2021; Zhu et al., 2021). Eight weeks after puncture, all rats were subjected to magnetic resonance imaging (MRI) and subsequently sacrificed for histopathologic analysis.

### MRI Evaluation

MRI was performed 8 weeks after puncture. Sagittal T2-weighted images were obtained for assessment of signal as well as disc structural changes using a 3.0 T MRI scanner (Siemens AG, Erlangen, Germany). Settings for the scanner were as previously reported (Xu et al., 2019). MRI results were assessed by a blinded orthopedics researcher who used the IDD classification system reported by Pfirrmann et al. (2001).

### Histological Analysis

IVD tissue was fixed in buffered paraformaldehyde (4%), decalcified for 4 weeks in EDTA, paraffin-embedded after which three serial sections of 4 µm were obtained. Hematoxylin and eosin (H and E) and safranin-O (S-O) were used to stain the sections.

### Statistical Analysis

Data are shown as mean ± SD. Statistical analyses were conducted using GraphPad Prism 6.0 (GraphPad Software, United States). Differences between and among groups were evaluated by the Student’s t-test and analysis of variance (ANOVA), respectively. Differences were considered statistically significant when *p* < 0.05.

## Results

### Effects of EGCG and H_2_O_2_ on the NPC Viabilities

**FIGURE 1:**
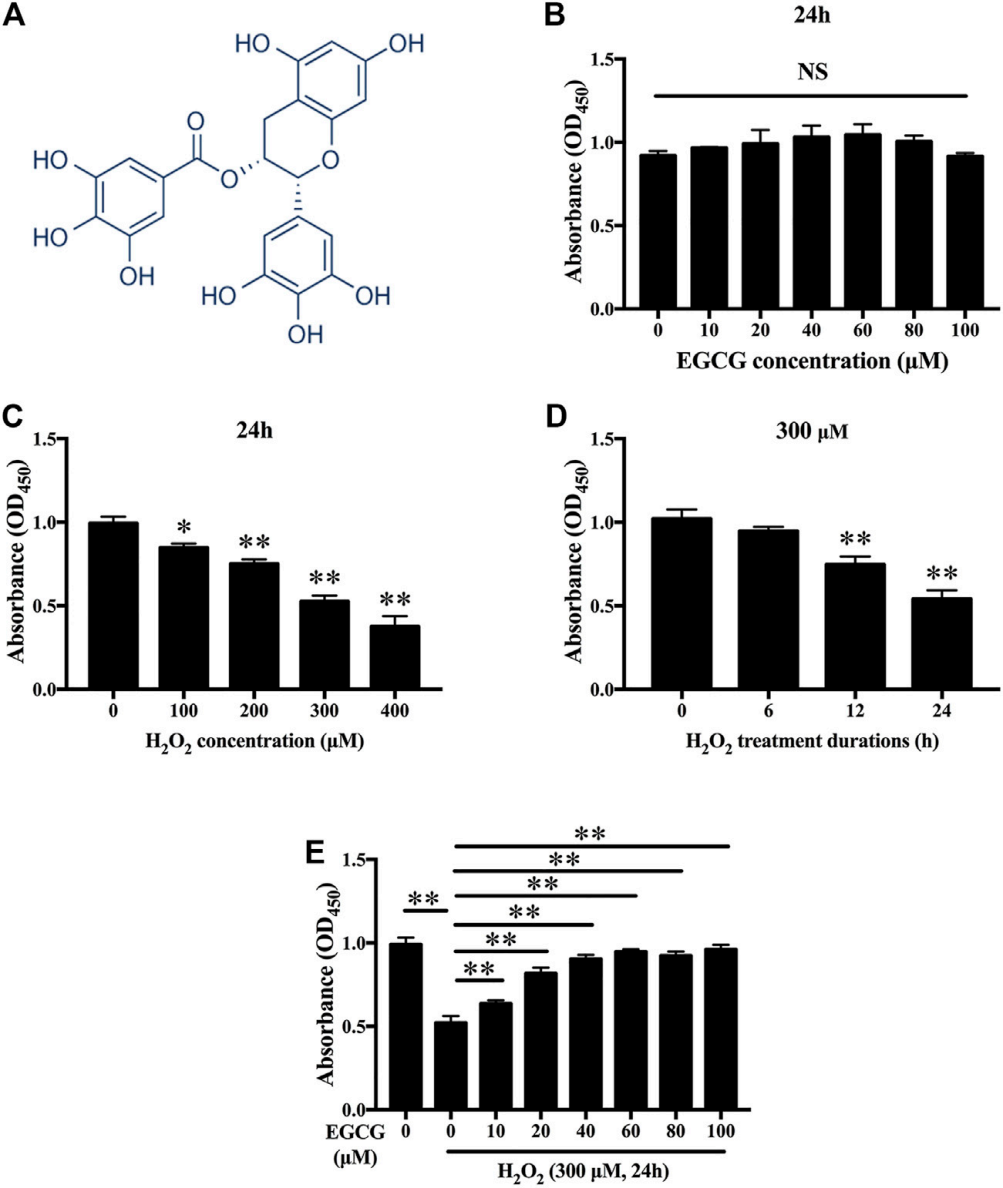
Effects of EGCG as well as H_2_O_2_ on NPCs viability. **(A)** Chemical structure of EGCG. **(B)** Findings from CCK-8 indicating NPCs viability after treatment with various concentrations of EGCG and **(C)** H_2_O_2_ for 24 h. **(D)** Cell viabilities of NPCs after 300 μM H_2_O_2_ treatment at various times (0, 6, 12, as well as 24 h). **(E)** CCK-8 findings indicating NPCs viability after treatment with 300 µM H_2_O_2_ and different concentrations of EGCG for 24 h. Data are shown as mean ± SD (*n* = 3). ^*^ *p* < 0.05, ^**^ *p* < 0.01.

Figure 1A shown the chemical structure for EGCG. To establish the effects of EGCG and H_2_O_2_ on NPC viability, cells were treated with varying concentrations of EGCG or H_2_O_2_ for 24 h, after which viability was assessed by the CCK-8 assay. Figure 1B showed that EGCG had no significant cytotoxic effects on NPCs at concentrations of up to 100 μM H_2_O_2_ dose-dependently decreased the viability of the NPCs (Figure 1C). Then, 300 µM H_2_O_2_ was chosen for the *in vitro* models. As shown in Figure 1D, short-term treatment with H_2_O_2_ (6 h) had almost no impact on the viability of NPCs, while prolonged treatment with H_2_O_2_ (12 and 24 h) significantly reduced NPC viability. EGCG exerted an obvious protective outcome against H_2_O_2_-mediated cell death, with 40 µM EGCG exerting the best therapeutic effects (Figure 1E).

### EGCG Attenuates H_2_O_2_-Evoked Extracellular Matrix (ECM) Degradation in NPCs

**FIGURE 2:**
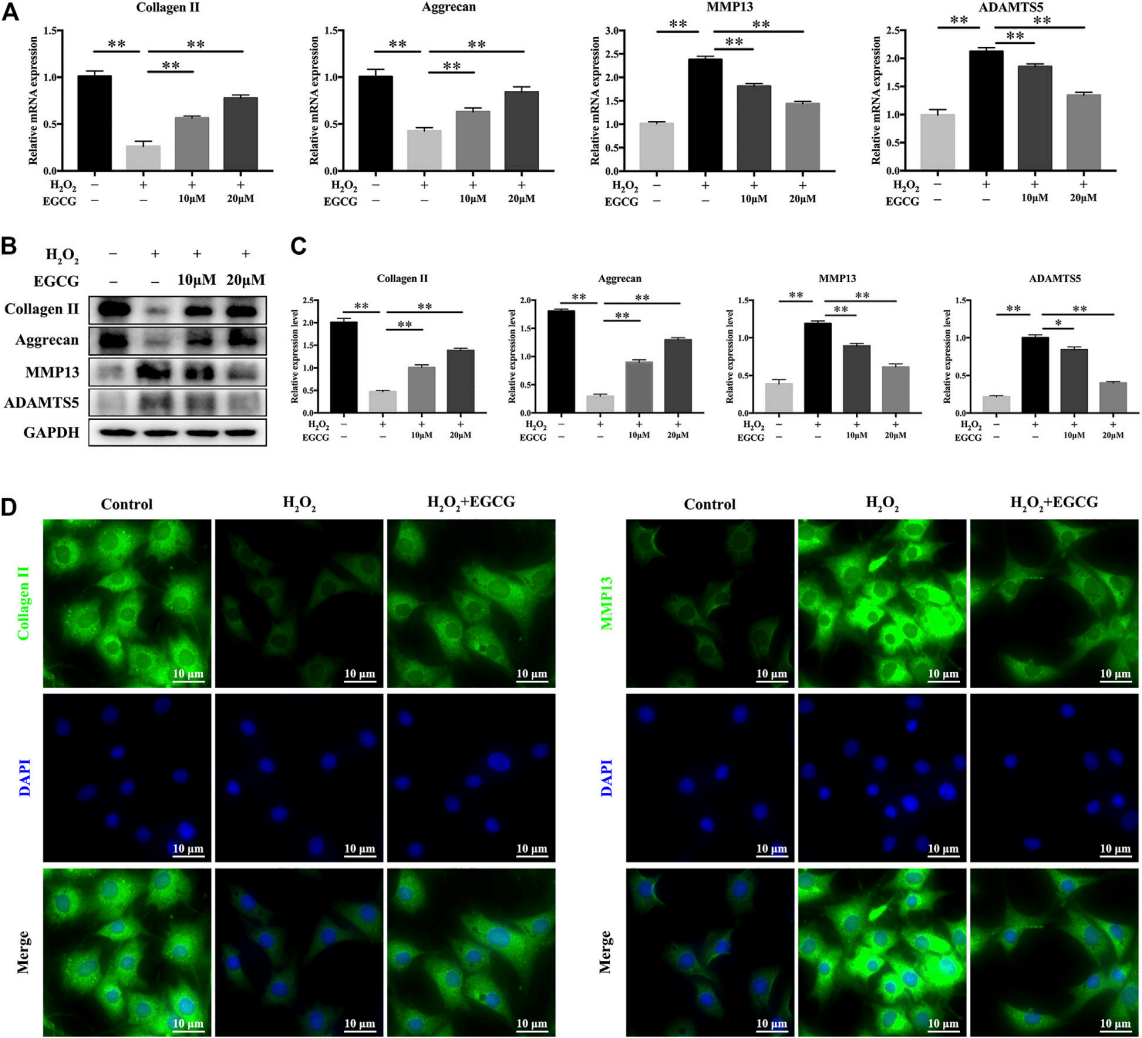
EGCG attenuates H_2_O_2_-induced ECM degradation in NPCs. **(A)** NPCs were pretreated with EGCG for 4 h and then stimulated with H_2_O_2_ for 24 h, qPCR results showing expression levels of Collagen II, Aggrecan, MMP13, and ADAMTS5. **(B)** Western blot findings of protein expression levels. **(C)** Quantifications of signal intensities. GAPDH was the loading control. **(D)** Immunofluorescence of Collagen II as well as MMP13 in NPCs. Data are shown as mean ± SD (*n* = 3). ^*^ *p* < 0.05, ^**^ *p* < 0.01.

Next, we examined the effects of EGCG on H_2_O_2_-mediated mRNA as well as protein expression levels of Aggrecan, Collagen II, ADAMTS5 and MMP13 in NPCs. H_2_O_2_ stimulation notably downregulated the mRNA expression of Aggrecan and Collagen II in NPCs and upregulated MMP13 and ADAMTS5 mRNAs. Pretreatment with EGCG significantly enhanced Collagen II and Aggrecan mRNA expression and inhibited the mRNA expression levels of ADAMTS5 and MMP13 (Figure 2A). In line with the mRNA expression results, western blot confirmed that ADAMTS5 and MMP13 protein levels were remarkably inhibited by pretreatment with EGCG, notably alleviating ECM degeneration (Figures 2B,C). Moreover, immunofluorescence confirmed that the expression levels of MMP13 and Collagen II were consistent with the qPCR and western blot results (Figure 2D). Therefore, EGCG exerts its protective effects by inhibiting the expression of ECM-degrading proteases, upregulating anabolic factor expression, and restoring the balance between anabolic as well as catabolic processes in NPCs under oxidative stress.

### EGCG Inhibits H_2_O_2_-Induced NPC Apoptosis

**FIGURE 3:**
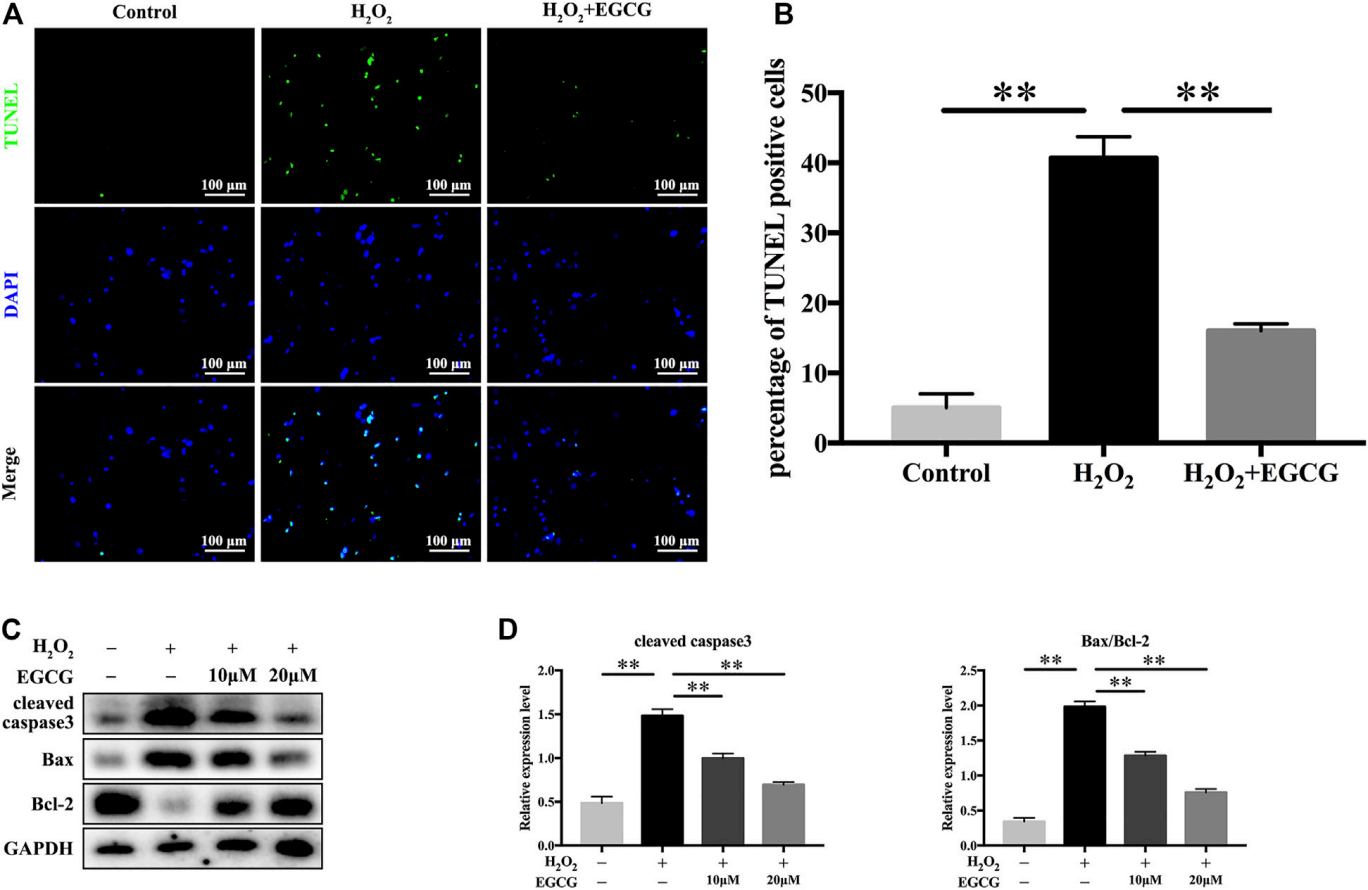
EGCG inhibits H_2_O_2_-induced NPCs apoptosis. **(A)** NPC apoptosis levels in every treatment group was measured by TUNEL assay, and **(B)** TUNEL-positive cells were quantified. **(C)** Western blot assay for cleaved caspase3, Bax, as well as Bcl-2 expression levels following treatment with varying EGCG doses (with or without 300 μM H_2_O_2_ treatment) for 24 h. **(D)** Levels of various signal intensities. GAPDH was the loading control. Data are shown as mean ± SD (*n* = 3). ^*^ *p* < 0.05, ^**^ *p* < 0.01.

Further, we examined the antiapoptotic effects of EGCG under H_2_O_2_ stimulation. TUNEL analysis revealed a higher rate of apoptosis in 300 μM H_2_O_2_-treated NPCs when compared to control NPCs, but pretreatment with EGCG significantly ameliorated H_2_O_2_-induced apoptosis (Figures 3A,B). Western blot analysis showed elevated levels of the apoptotic protein cleaved caspase3 and the apoptosis-related Bax/Bcl-2 ratio after H_2_O_2_ treatment; however, these increases were inhibited by pretreatment with EGCG (Figures 3C,D). These results are comparable to TUNEL staining results.

### Effects of EGCG on Circadian Misalignment in H_2_O_2_-Exposed NPCs

**FIGURE 4:**
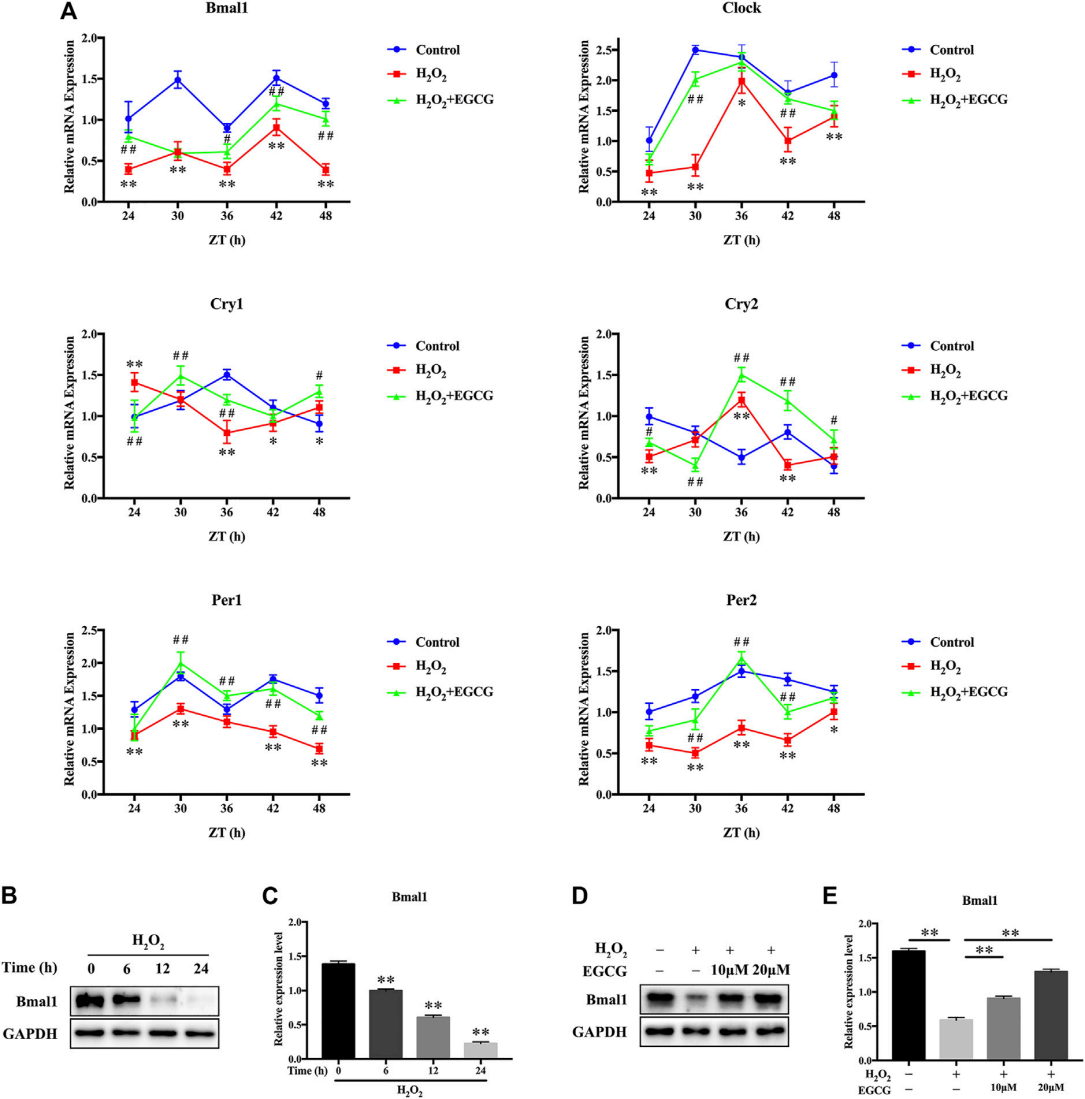
Effects of EGCG against circadian misalignment in NPCs after exposure to H_2_O_2_. After 2 h of serum shock, NPCs were pretreated with 20 μM EGCG for 12 h after which they were treated with 300 μM H_2_O_2_ for 12 h. Then, cells were obtained for mRNA analysis between the 24 and 48 h at 6 h intervals. **(A)** mRNA expression levels of the circadian oscillator constituents Bmal1, Clock, Cry1, Cry2, Per1, as well as Per2 in NPCs. Transcription levels were evaluated by qPCR followed by normalization to GAPDH mRNA levels. ZT, Zeitgebers (German for “time giver”). ^*^ *p* < 0.05, ^**^ *p* < 0.01, indicates H_2_O_2_ vs control. ^#^ *p* < 0.05, ^##^ *p* < 0.01, indicates H_2_O_2_ + EGCG vs H_2_O_2_. **(B)** The Bmal1 expression changes after H_2_O_2_ stimulation for different times were evaluated by western blot analysis, and GAPDH was the loading control. **(C)** Results of densitometric analyses of the blots. **(D)** NPCs were pretreated with varying EGCG doses for 4 h after which they were stimulated for 24 h using H_2_O_2_. The effects of EGCG on H_2_O_2_-triggered Bmal1 expression changes were determined by western blot analysis, and GAPDH was the loading control. **(E)** The results of densitometric analyses of the blots. The data are shown as mean ± SD (*n* = 3). ^*^ *p* < 0.05, ^**^ *p* < 0.01.

H_2_O_2_-mediated oxidative stress produces robust phase shifts in circadian clocks in a time-of-day-specific manner (Ranieri et al., 2015). To investigate the effects of EGCG on circadian clock temporal regulation, we measured mRNA oscillations of essential clock components in NPCs after H_2_O_2_ treatment. After serum shock for 2 h, NPCs were treated with 20 μM EGCG for 12 h after which they were treated for 12 h with 300 μM H_2_O_2_. Then, mRNA analysis was performed between 24 and 48 h at 6 h intervals. Figure 4A showed that, in the control group, the transcription levels of clock genes exhibited diurnal variations. Oscillatory amplitudes of Bmal1, Clock, as well as clock-related genes (Per1, Per2) were dramatically dampened in H_2_O_2_-treated rat NPCs compared with control NPCs. Additionally, H_2_O_2_ provoked phase shifts in the expression rhythms of circadian clock components (Cry1, Clock, Cry2). Intriguingly, EGCG pretreatment reversed the daily oscillations and phase shifts of the circadian clock induced by H_2_O_2_, increasing the trough/peak ratios of mRNA expression levels (Figure 4A).

To establish the importance of Bmal1, a core circadian clock gene, in H_2_O_2_-induced oxidative damage in NPCs, western blot analyses were conducted. The expression levels of Bmal1 were gradually suppressed after H_2_O_2_ treatment (Figures 4B,C). Furthermore, a rescue assay showed that after EGCG treatment, the protein expression levels of Bmal1 gradually recovered (Figures 4D,E). These results suggest that EGCG may restore the circadian clock in NPCs under H_2_O_2_ stimulation.

### Bmal1 Plays an Essential Role in EGCG-Mediated Alleviation of ECM Degradation in NPCs Under Oxidative Stress Conditions

**FIGURE 5:**
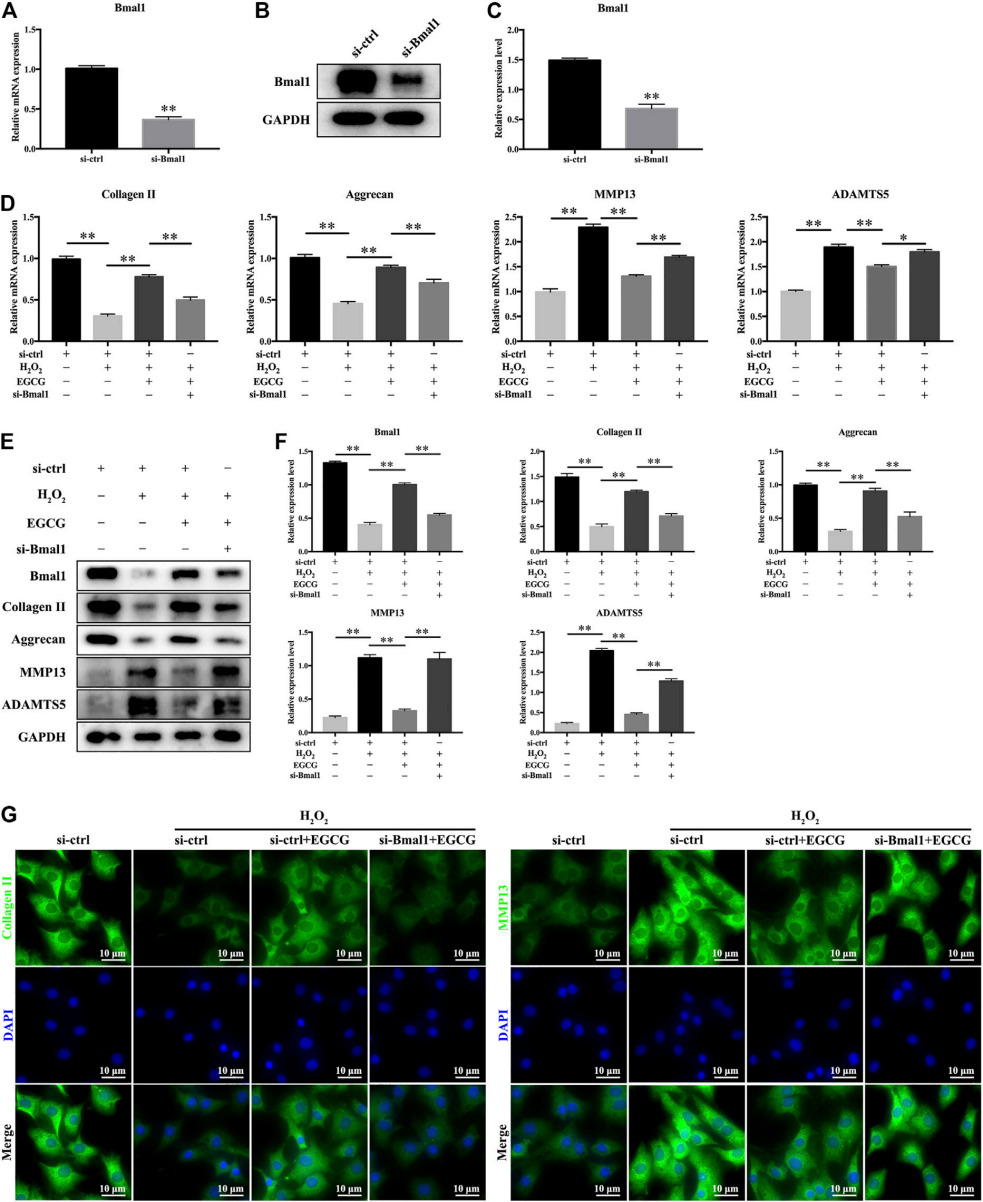
Bmal1 is involved in EGCG-mediated prevention of ECM degradation in NPCs under oxidative stress conditions. Rat NPCs were transfected with si-Bmal1 or si-ctrl for 48 h, followed by pretreatment with or without 20 μM EGCG for 4 h and with H_2_O_2_ for an additional 24 h **(A)** mRNA and **(B,C)** protein levels of Bmal1. **(D)** mRNA levels of Collagen II, Aggrecan, MMP13, and ADAMTS5. **(E)** The expression levels of Bmal1, Collagen II, Aggrecan, MMP13, and ADAMTS5 were examined in rat NPCs using western blot. **(F)** Findings of densitometric analysis of the blots are shown. **(G)** Immunofluorescence of Collagen II and MMP13 in NPCs. Data are shown as mean ± SD (*n* = 3). ^*^ *p* < 0.05, ^**^ *p* < 0.01.

To investigate the effect of Bmal1 on IVD homeostasis, siRNAs were used to knock down Bmal1 in rat NPCs. Figures 5A–C showed that after si-Bmal1 transfection, mRNA as well as protein expression levels of Bmal1 were much lower than those in si-ctrl-transfected cells. As revealed in Figures 5D–F, EGCG pretreatment attenuated the H_2_O_2_-evoked changes in the mRNA as well as protein expression levels of Bmal1, Collagen II, Aggrecan, MMP13 and ADAMTS5 in NPCs, while this effect was partially ameliorated by si-Bmal1. That is to say, Figures 5D–F showed that Bmal1 knockdown significantly impaired EGCG-alleviated ECM degradation under oxidative stress conditions in rat NPCs. Furthermore, the immunofluorescence results described that the fluorescence intensities of Collagen II as well as MMP13 were consistent with qPCR and western blot findings (Figure 5G).

### EGCG Ameliorates H_2_O_2_-Triggered Apoptosis in Rat NPCs by Modulating the Circadian Clock

**FIGURE 6:**
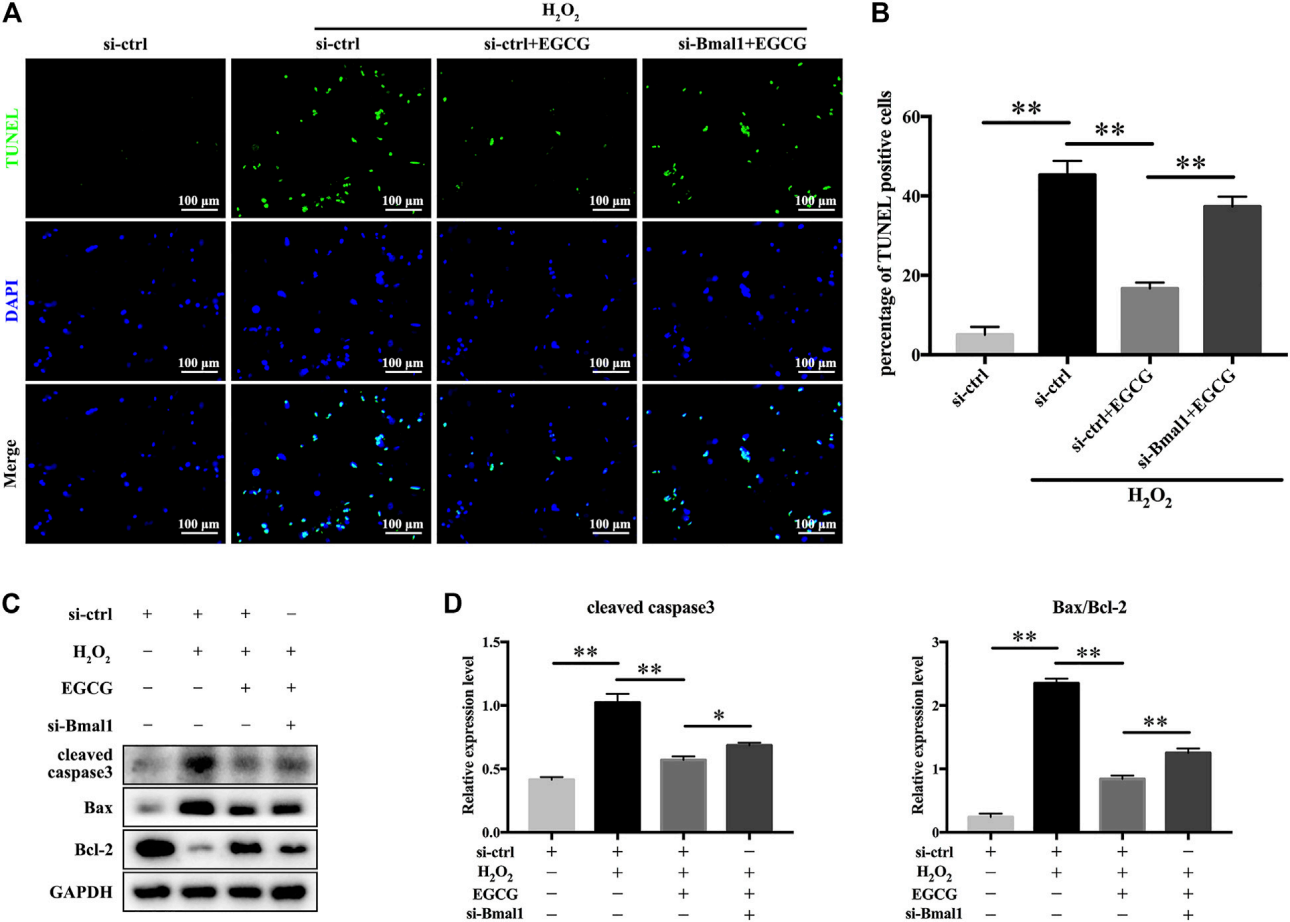
EGCG attenuates H_2_O_2_-induced apoptosis in a Bmal1-dependent manner in rat NPCs. Rat NPCs were transfected with si-Bmal1 or si-ctrl for 48 h, followed by pretreatment with or without 20 μM EGCG for 4 h and with H_2_O_2_ for an additional 24 h. **(A)** Apoptosis of NPCs in each treatment group was examined by TUNEL assay, and **(B)** the TUNEL-positive cells were quantified. **(C)** Cleaved caspase3, Bax as well as Bcl-2 levels were measured by western blot analysis. **(D)** Quantification of the blots. Data are shown as the mean ± SD (*n* = 3). ^*^ *p* < 0.05, ^**^ *p* < 0.01.

To further explore whether Bmal1 participated in the EGCG-mediated amelioration of NPC apoptosis, TUNEL staining was performed, and apoptosis-related proteins were monitored. As shown in Figure 6A, EGCG pretreatment significantly reduced the increase in apoptosis provoked by H_2_O_2_, but downregulation of Bmal1 markedly crippled the antiapoptotic effect of EGCG (Figure 6B). Furthermore, the apoptotic protein cleaved caspase3 and the apoptosis-related Bax/Bcl-2 ratio in rat NPCs were substantially elevated in the si-Bmal1 treatment group compared with the EGCG group (Figures 6C,D). Collectively, these results indicate a Bmal1-dependent efficacy of EGCG against apoptosis in NPCs under oxidative stress conditions.

### EGCG Treatment Ameliorated IDD in a Needle Puncture Rat Model by Regulating the Circadian Clock

**FIGURE 7:**
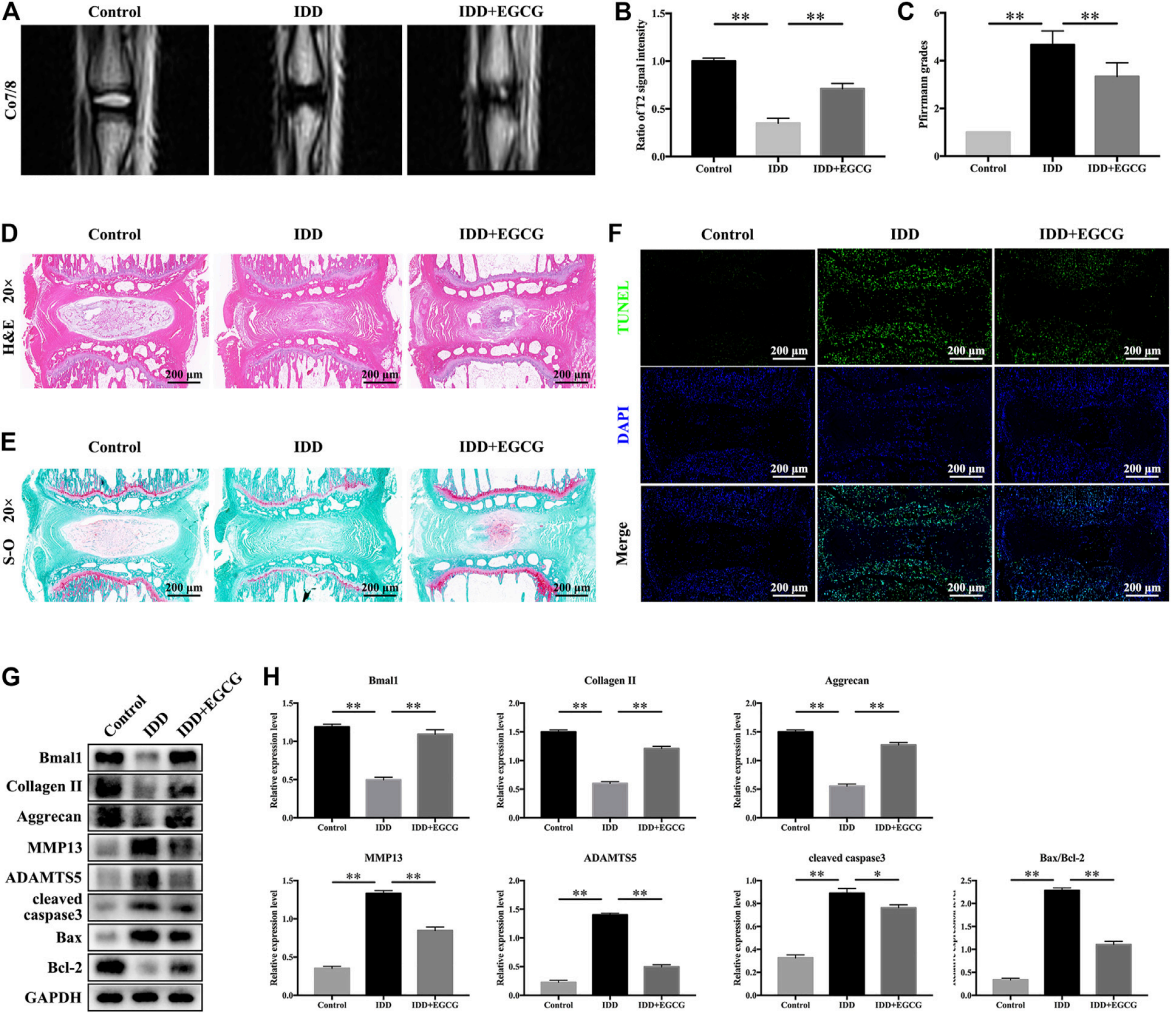
EGCG alleviated IDD progression in a rat model by regulating the circadian clock. **(A)** T2-weighted MRI images of rat tails at 8 weeks after surgery. **(B)** Quantitative analysis of the T2 disc signal intensity. **(C)** Pfirrmann MRI grades for the three groups at 8 weeks post-surgery. **(D)** Representative H and E as well as **(E)** S-O staining of the punctured discs from the various groups at 8 weeks after surgery. **(F)** A TUNEL assay was performed conducted to assess apoptotic levels in NP samples from discs in various groups. **(G)** Western blot assay was conducted, and **(H)** protein levels of Bmal1, Collagen II, Aggrecan, MMP13, ADAMTS5, cleaved caspase3 and Bax/Bcl-2 in the NP samples of rats are shown. Data are shown as mean ± SD (*n* = 12). ^*^ *p* < 0.05, ^**^ *p* < 0.01.

According to the *in vitro* experimental results, we evaluated the effects of EGCG *in vivo* in an IDD rat model established by tail needle puncture. To evaluate the degree of disc degeneration, we conducted MRI and assessed Pfirrmann grade scores at 8 weeks after puncture in various groups. Figure 7A showed that after needle puncture, the IVDs in the IDD group exhibited lower T2-weighted signal intensities and higher Pfirrmann grades than those in the control group. However, with EGCG treatment, the IVDs in the IDD + EGCG group exhibited elevated T2-weighted signal intensity values and low Pfirrmann grades than those in the IDD group (Figures 7A–C). The protective effects of EGCG *in vivo* were corroborated by H and E and S-O staining of rat IVD tissues. H and E staining revealed that IVDs in the control group had complete NP tissue and that the NPCs were equally dispersed in the ECM. Annulus fibrous (AF) tissue structures around the NP were clear and exhibited good continuities with NP tissues (Figure 7D). Compared to the control group, NP tissues from rats in the IDD group were significantly atrophied and exhibited poor continuity with AF; moreover, AF tissue structures were destroyed. However, EGCG treatment markedly suppressed the destruction of IVD structures as well as the NP tissue fibrosis (Figure 7D). S-O staining revealed that the amount of red (positive) tissues representing the proteoglycan matrix in the IDD group was significantly lower than that of the control group. However, compared with the IDD group, the EGCG treatment group had partial retention of these tissues (Figure 7E).

To detect apoptosis in IVD tissue in various treatment groups, we conducted a TUNEL assay. The fluorescence intensity of apoptosis in IVD was elevated in the IDD group when compared to the control group, but markedly suppressed in the EGCG treatment group when compared to the IDD group (Figure 7F). Moreover, western blot analysis revealed that EGCG treatment elevated clock protein Bmal1, as well as Aggrecan and Collagen II levels, and decreased MMP13, ADAMTS5, cleaved caspase3 levels and the Bax/Bcl-2 ratio (Figures 7G,H), which is consistent with our *in vitro* findings. In conclusion, these data indicate that EGCG improves core circadian clock protein Bmal1 expression and ameliorates IDD *in vivo*.

## Discussion

Lower back pain (LBP), which is an increasingly common disorder, reduces patients’ quality of life and places economic burdens on healthcare systems and society. IDD is generally considered to be the main cause of LBP (Lyu et al., 2021). At present, patients with IDD are prescribed non-steroidal anti-inflammatory drugs or muscle relaxants to relieve symptoms. However, these drugs are unable to effectively prevent IDD or alleviate its progression. Previous studies have shown that the most important features of IDD is the apoptotic of NPCs (Xu et al., 2019) and degradation of the extracellular matrix (ECM). The main components of ECM were Collagen II and Aggrecan, and the degradation of which was closely related to the activity of ADAMTS5 and MMP13 (Zhang et al., 2021). Our results indicated that EGCG attenuates H_2_O_2_-induced ECM degradation and apoptosis in NPCs (Figures 2, 3). Recent studies have shown that there is an endogenous circadian clock in IVD tissue, which is synchronized with 24 h to control key aspects of IVD physiology (Dudek et al., 2017; Morris et al., 2021). In mice, the disruption of this molecular time-keeping mechanism can lead to premature aging and degeneration of the IVD, which indicates that the circadian clock is a key regulator of IVD tissue function (Dudek et al., 2017).

Some dietary ingredients in food have bioactivities that affect the circadian rhythm of the human body, and some specific nutrients and food factors can regulate the circadian clock of cells or the circadian clock system of the entire body, to restore normal rhythms (Ribeiro et al., 2021). Chicoric acid (CA) is regarded as a nutraceutical with powerful antioxidant and antiobesity activities. Previous studies revealed that CA can improve the misalignment and the relatively shallow daily oscillations of clock genes and protect the lipid metabolism dysfunction via linking the circadian regular Bmal1 (Guo et al., 2018). Nobiletin is a polymethoxylated flavone found in certain citrus fruits, and exerts reprograming of the circadian clock properties, partially reverse the relatively shallow daily oscillations of circadian clock genes and reset phase-shifting circadian rhythms in primary hepatocytes under metabolic disorders conditions (Qi et al., 2018a). Qi et al. (2018b) demonstrated that tea polyphenols efficiently reverse the relatively shallow daily oscillations of the clock genes triggered by H_2_O_2_ in hepatic HepG2 cells, with an increase in the trough peak ratios of the mRNA levels. Besides, EGCG has been shown to target clock genes, thereby exerting its health effects (Li et al., 2016; Mi et al., 2017a; Mi et al., 2017b). It is unclear whether Bmal1 plays a role in preventive effects of EGCG on IDD. Moreover, molecular mechanisms of circadian clock-promoting effects of EGCG against IDD have not been elucidated. We found that EGCG pretreatment significantly alleviated the processes of circadian misalignment, including circadian amplitude dampening as well as phase shifts, in H_2_O_2_ treated rat NPCs (Figure 4). To confirm the function of EGCG *in vivo*, we further observed its effect in a rat model. Consistent with the above reports, EGCG reduced apoptosis-related proteins (cleaved caspase3, Bax/Bcl-2 ratio) and catabolic enzymes (MMP13 and ADAMTS5), enhanced Bmal1 and ECM compositions (Collagen II, Aggrecan) in the rat NP tissues (Figures 7G,H), thereby blocked the progression of IDD induced by IVD puncture.

EGCG, which is a catechins monomer isolated from tea, is the main component of green tea polyphenols. It was reported that EGCG plays important antioxidant, anti-carcinogenic, anti-microbial, and neuroprotective effects (Chakrawarti et al., 2016). In a previous case control, it was found that tea drinking habit is a protective factor for IDD (Deng et al., 2017). Previous studies has been investigated the effect of EGCG on the oxidative stress and inflammatory response in intervertebral disc in animal model and *in vitro* cell culture. One study has provided results that EGCG treatment demonstrated significant protective effects in cell viability, apoptosis, cell cycle arrest and inflammatory status under oxidative stress through down-regulation of cGAS/Sting/NLRP3 pathway (Tian et al., 2020). Findings from another study suggest that EGCG significantly inhibited the expression of pro-inflammatory mediators and matrix metalloproteinases *in vitro*, as well as radiculopathic pain *in vivo*, most probably by modulation of the activity of IRAK-1 and its downstream effectors p38, JNK and NF-κB (Krupkova et al., 2014). Based on the promising effects of EGCG and critical role of circadian clock in the pathological progress of IDD, we conducted an experiment to investigate the effects of EGCG on circadian misalignment under oxidative stress in IDD. Although we confirmed the protective effect of EGCG on NPCs (Figures 1–3), our results differ somewhat from several prior researches. There are four novel findings in our study. Firstly, we finding that EGCG pretreatment reversed the daily oscillations and phase shifts of the circadian clock induced by H_2_O_2_, increasing the trough/peak ratios of clock gene expression levels (Figure 4A). Secondly, H_2_O_2_ suppressed the core clock gene Bmal1 protein expression in a time-dependent manner (Figures 4B,C), but EGCG treatment altered this conditions (Figures 4D,E). Thirdly, our findings further suggest that Bmal1 plays an essential role in EGCG-mediated alleviation of ECM degradation and apoptosis in NPCs under oxidative stress (Figures 5, 6). Finally, EGCG treatment ameliorated IDD and improved Bmal1 expression *in vivo* (Figure 7). These novel findings in our experiments enrich the candidate drug targets of EGCG, indicating that the application of EGCG might be a plausible strategy for the treatment of degenerative diseases related to the circadian clock disorders, such as IDD.

During the progression of IDD, oxidative stress in IVD microenvironments accelerate disc degeneration, leading to matrix-degrading proteases to degrade ECM (Zhang et al., 2021). In addition, increasing evidence has shown that there is an interaction between oxidative stress and circadian clock as alterations in redox status can have an effect on core clock functions, while clock proteins control cell redox homeostasis (Ranieri et al., 2015; Liu et al., 2021). Binding of Clock/Bmal1 to DNA depends on the ratio of NAD(H)/NADP(H), with improved binding observed reducing circumstances (Rutter et al., 2001). Circadian clocks in H_2_O_2_ levels were observed in cultured cells and mouse livers, which directly control the rhythms of Clock function through oxidation of cysteine (Pei et al., 2019). The protein p66^shc^, which is redox-responsive, is rhythmic, and its deletion interrupts Clock-associated oxidation rhythms. Moreover, its deletion changes transcription rhythms and behavior of mice (Pei et al., 2019). The pentose phosphate pathway (PPP) is essential for NADPH production to promote ROS generation through NADPH oxidase enzymes, and for glutathione generation, PPP inhibition can also alter clock function (Rey et al., 2016). PPP suppression with resulting loss of NADPH increases oxidative stress, activates the Nrf2 redox response pathway, promotes Clock/Bmal1 DNA binding, changes in clock gene expression levels, and extension of the circadian clock period (Rey et al., 2016). Nrf2 itself seems to regulate the function of the clock. *Nrf2* ^*−/−*^ cells have reduced clock gene expression levels and weakened circadian rhythms of Per2 expression (Wible et al., 2018). Therefore, oxidative stress regulates circadian rhythm functions through various mechanisms.

In contrast, the core clock regulates redox response gene expression and determines cell responses in oxidative stress conditions. In ROS sensitive environments, *Drosophila* perform circadian rhythms, while in arrhythmic *Per* ^*01*^ mutant flies, this sensitivity is lost. *Per* ^*01*^ flies exhibited shortened life expectancy, aggravated oxidative damage as well as age-associated neuronal degenerations (Krishnan et al., 2009; Krishnan et al., 2012). At multiple levels, glutathione, an essential cellular antioxidant, is clock regulated. In *Drosophila* and rat, glutathione-producing enzymes, glutathione levels, and transferase enzymes all exhibit circadian oscillations (Beaver et al., 2012; Naseri Kouzehgarani et al., 2020). Nrf2 strongly regulates the synthesis of glutathione and is a transcriptional target for Bmal1 (Early et al., 2018). Bmal1 regulates *Nrf2* in macrophages (Early et al., 2018), pancreatic *β* cells (Lee et al., 2018), and lung cells (Pekovic-Vaughan et al., 2014), while diminished Bmal1 causes dampened Nrf2-associated antioxidant responses as well as elevated ROS levels. Therefore, the loss of Bmal1 enhances oxidant injury in several organs (Xie et al., 2020). REV-ERBα, a clock regulating protein, can be provoked by elevated ROS levels, thereby regulating the antioxidant transcription factor FOXO1 expression, and stimulating mitochondrial biogenesis and autophagy (Woldt et al., 2013; Yang et al., 2014; Sengupta et al., 2016; Ji et al., 2019). Overexpression of REV-ERBα can improve mitochondrial function and provide protection against oxidative stressors (Woldt et al., 2013; Sengupta et al., 2016; Ji et al., 2019). In *Drosophila melanogaster*, oxidative stress can reprogram genome-wide circadian transcriptions, promoting redox stress-associated responses (Kuintzle et al., 2017). In summary, the circadian clock responds to changes in cellular redox and regulates redox pathway expression. Since oxidative stress is highly correlated with various aspects of aging, age-associated changes in circadian clock function will accelerate oxidative injury.

We found that H_2_O_2_ significantly suppresses oscillatory amplitudes and induces phase shifts in clock genes (Bmal1, Clock) as well as clock-associated genes (Per1, Cry1, Cry2, Per2) in NPCs. Interestingly, EGCG pretreatment dramatically reversed oxidative stress-associated circadian clock disorders. It is probable that circadian misalignment induced by H_2_O_2_ leads to a vicious cycle with elevated ROS production followed by oxidative stress exacerbation and circadian disorders. Accordingly, treatment with EGCG may be a novel intervention method to break this vicious cycle, thereby leading to oxidative stress as well as circadian asynchrony through regulating Bmal1. This study informs on future applications of EGCG as a novel therapeutic strategy for IDD through involving circadian clock-related mechanisms.

## Conclusion

In conclusion, our study reveals EGCG can serve as a natural circadian clock modulator that protects NPCs against apoptosis and ECM degradation under oxidative stress in a Bmal1-dependent manner. It is our hope that the present work will spur future application of EGCG as a Bmal1-targeting compound to mitigate redox status imbalance and circadian rhythm disorders-related pathologies, such as IDD.

## Data Availability

The original contributions presented in the study are included in the article, further inquiries can be directed to the corresponding authors.
